# Bridging Glycomics and Genomics: New Uses of Functional Genetics in the Study of Cellular Glycosylation

**DOI:** 10.3389/fmolb.2022.934584

**Published:** 2022-06-16

**Authors:** Natalie Stewart, Simon Wisnovsky

**Affiliations:** ^1^ Biochemistry and Microbiology Dept, University of Victoria, Victoria, BC, Canada; ^2^ Faculty of Pharmaceutical Sciences, University of British Columbia, Vancouver, BC, Canada

**Keywords:** glycomics, genomics, CRISPR, RNA-seq, ChIP-seq, CRISPR screen, sc-RNA seq

## Abstract

All living cells are coated with a diverse collection of carbohydrate molecules called glycans. Glycans are key regulators of cell behavior and important therapeutic targets for human disease. Unlike proteins, glycans are not directly templated by discrete genes. Instead, they are produced through multi-gene pathways that generate a heterogenous array of glycoprotein and glycolipid antigens on the cell surface. This genetic complexity has sometimes made it challenging to understand how glycosylation is regulated and how it becomes altered in disease. Recent years, however, have seen the emergence of powerful new functional genomics technologies that allow high-throughput characterization of genetically complex cellular phenotypes. In this review, we discuss how these techniques are now being applied to achieve a deeper understanding of glyco-genomic regulation. We highlight specifically how methods like ChIP-seq, RNA-seq, CRISPR genomic screening and scRNA-seq are being used to map the genomic basis for various cell-surface glycosylation states in normal and diseased cell types. We also offer a perspective on how emerging functional genomics technologies are likely to create further opportunities for studying cellular glycobiology in the future. Taken together, we hope this review serves as a primer to recent developments at the glycomics-genomics interface.

## Introduction

Glycosylation is a common property of all cellular life. In human cells, a number of different monosaccharides can be covalently connected to produce a range of extended, branched oligosaccharides called glycans. These molecules can then be covalently linked to discrete asparagine (*N*-linked glycosylation) or serine/threonine (O-linked glycosylation) residues located on secreted or cell-surface proteins. Glycans can also be directly attached to membrane-embedded lipid molecules (Varki A, 2015–2017). Specific glycans serve as ligands for a range of carbohydrate-binding signaling receptors, which play important roles in regulating the activation and differentiation of immune cells (Thiemann and Baum, 2016; Brown et al., 2018; Duan and Paulson, 2020; Smith and Bertozzi, 2021). Like other post-translational modifications, glycosylation can also act as a molecular switch that modulates the binding of signaling receptors to their ligands (Gee et al., 2003; Li et al., 2016). Finally, glycans can mediate complex, multimolecular interactions that broadly regulate cell-surface trafficking and residency of glycosylated proteins. These molecules thus profoundly influence all aspects of cell signaling and metabolism (Demetriou et al., 2001; Lau et al., 2007; Lau and Dennis, 2008).

**FIGURE 1:**
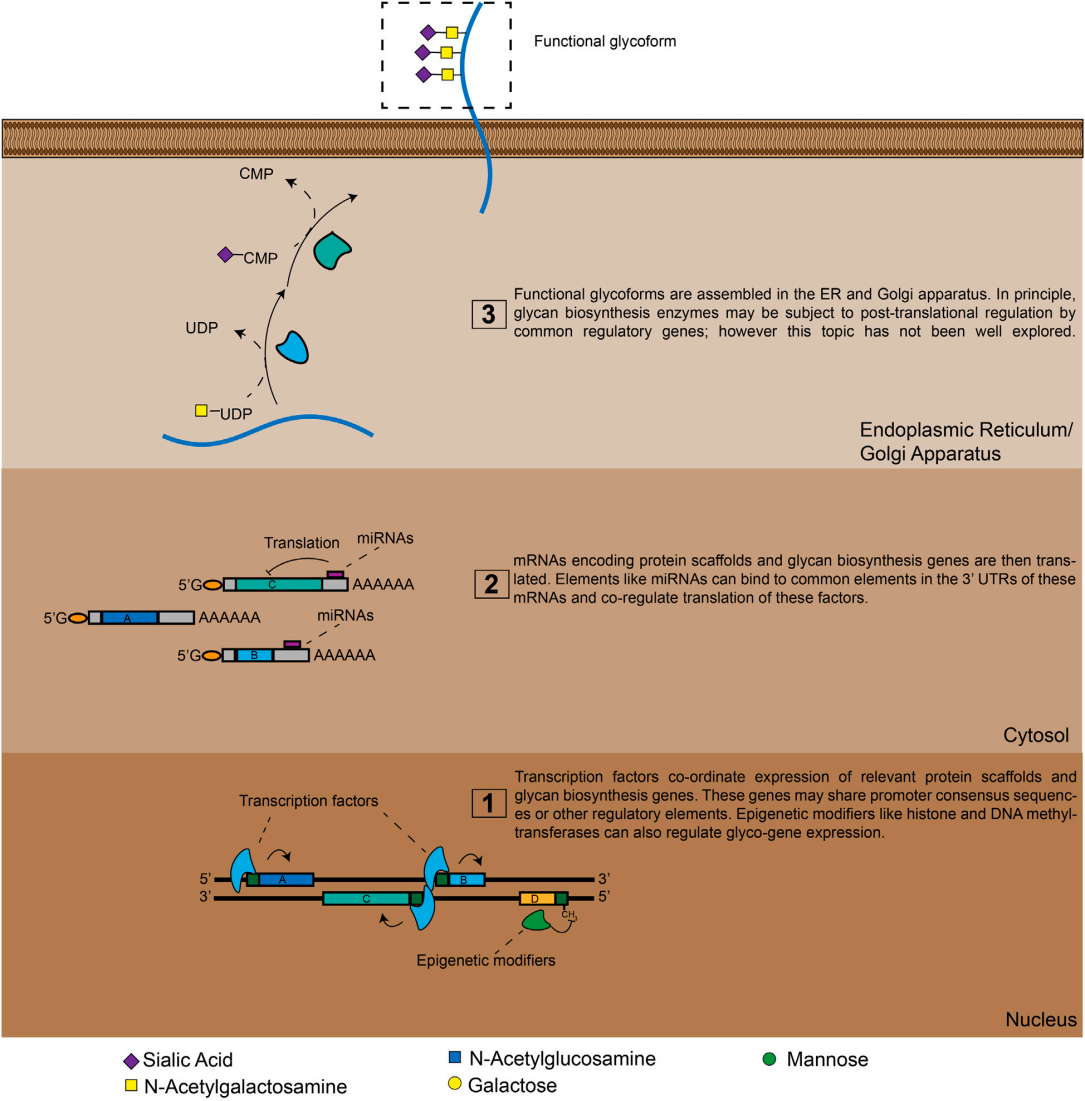
A high-level view of glyco-genomic regulation. Cell-surface glycosylation patterns emerge from the expression and/or repression of many enzymes. TFs and epigenetic modifiers can co-ordinate these polygenetic circuits at the transcriptional level, while miRNAs do so at the translational level. Additional layers of regulation are also possible, meaning that a cell’s glycomic state can only be imperfectly predicted by analyzing mRNA and miRNA expression levels.

Like proteins, glycans are functional biopolymers made up of discrete, modular building blocks. However, unlike proteins, the information required to make a specific glycan is not directly encoded in the DNA sequence of a corresponding gene. Glycans are instead produced through complex circuits that involve co-ordinated expression of many different genes (Figure 1). At the most basic level, glycans are constructed by biosynthetic enzymes like glycosyltransferase (GT). These factors build glycan chains by selectively attaching monosaccharides to specific underlying oligosaccharide substrates (Figure 1) (Varki A, 2015; Moremen and Haltiwanger, 2019). In the human genome there are hundreds of genes that encode GTs with different underlying substrate specificities (Narimatsu et al., 2018). The set of glycan structures produced by a cell will thus be greatly influenced by which GT genes are actively expressed in that cell type (Varki A, 2015–2017). Additionally, the ability of glycans to perform specific functions can depend on the density and/or orientation in which glycans are presented on the cell-surface (Sako et al., 1995; Hashimoto et al., 2019). As a result, many glycans only acquire distinct functional properties when they are attached to a specific protein scaffold (Sako et al., 1995; Sackstein, 2011). Genes encoding these scaffolding molecules can therefore also be key components of glyco-regulatory genetic circuits (Figure 1). Finally, glycan biosynthesis is also broadly regulated by secondary mechanisms that tune glyco-ligand production through modulating GT localization and metabolic intermediate availability (Lau et al., 2007; Smith and Lupashin, 2008). GT is thus an emergent cellular property that arises from a staggeringly complex set of genetic interactions.

From this picture, a key question naturally arises: how are these polygenetic circuits regulated? As with other complex cellular processes, there are likely upstream “master regulators” that co-ordinate the biosynthesis of functional glycans by simultaneously modulating the activities of many different genes. In cases where aberrant glycosylation is involved in disease pathogenesis, these factors may serve as important disease biomarkers or novel therapeutic targets. Identifying new glyco-genetic circuits and the mechanisms that regulate their activity has thus always been a significant focus of glycoscience research. In particular, a defining trend of the past two decades has been a rapid increase in the reliability and throughput of various functional genomics methodologies and protocols. Instead of assessing gene function in a targeted, sequential way, researchers now can leverage next-generation sequencing (NGS) technology to routinely perform multiplexed analysis and/or perturbation of thousands of genes in a single biological experiment. Specific significant advances have included: 1) the progressive lowering of costs for whole-exome transcriptomic profiling by RNA-seq (Stark et al., 2019), 2) the methodical identification of transcription factor binding sites on a genome-wide scale by ChIP-seq (Wu et al., 2015), 3) functional genomic screening by shRNA and CRISPR/Cas9-based gene manipulation (Shalem et al., 2014), and 4) single cell gene expression profiling by single cell RNA-seq (scRNA-seq) (Yan et al., 2020; Nayak and Hasija, 2021). These advances have offered unprecedented opportunities for systematic dissection of genetically complex cellular phenotypes.

In the past few years, we have observed many new and exciting applications of these functional genetics methods towards the study of glycans in cell biology and disease. This trend of growing integration between glycomics and genomics appears to be accelerating. We believe research at this interface is poised to generate sweeping new insights into the regulation of glycosylation in human cells. In this review, we highlight some recent, significant developments in this area. While we have attempted to cover as much ground as possible, this discussion is not intended to be completely comprehensive. As these functional genomics techniques have been most broadly applied to mammalian cell models, we focus here on human rather than on microbial glycosylation. We also primarily discuss cell-surface carbohydrates rather than cytosolic (O-GlcNAc) glycosylation. While O-GlcNAc plays a fascinating set of roles in many biological processes, the biosynthesis of these glycans is subject to somewhat less complex genetic regulation when compared to cell-surface glycosylation (Yang and Qian, 2017). Our primary goal is to give glycobiology researchers a sense of how functional genomics techniques and resources can be usefully applied to general questions that may be of interest in their specific research areas. At the end of the article, we also discuss how new, leading-edge techniques may create further opportunities for glyco-genomics research in the future.

### Chromatin Immunoprecipitation Sequencing and RNA-Seq in Glycoscience: Identifying Master Regulators of Glycan Biosynthesis

Transcription factors (TFs) are key regulators of gene expression in mammalian cells. TFs usually bind to specific DNA sequence motifs found in gene promoter or enhancer regions (Pan et al., 2010; de Boer et al., 2020). Subsequent TF-mediated recruitment or blockade of factors like RNA polymerases can then either activate or repress gene expression (Pan et al., 2010; de Boer et al., 2020). Different TFs also interact with one another in complex gene regulatory networks, making the function of a given TF somewhat cell- and context-dependent (Jolma et al., 2015; Stampfel et al., 2015). By binding to regulatory regions in multiple genes with shared consensus sequences, specific TFs can simultaneously modulate expression of many factors within a specific biosynthetic pathway (Figure 1). This feature has always made them attractive targets for investigation as regulators of glycan biosynthesis (Guo and Pierce, 2015; Neelamegham and Mahal, 2016). TF(s) also participate in chromatin remodeling and DNA methylation, which has been shown to play a key role in long-term (epigenetic) transcriptional repression of various glycan biosynthesis genes (Bui et al., 2010; Greville et al., 2016; Indellicato and Trinchera, 2021).

Significant effort has thus been expended by a number of groups into identifying conserved TF-binding sites within the promoter regions of various GT enzymes. Historically, these studies had to be conducted on a targeted basis. Painstaking work was often required to sequence gene promoter regions and identify consensus sites for transcription factors that had known binding motifs. Investigators could also take the complementary approach. TFs of known importance could be overexpressed in cells and changes in expression of various glycogenes and/or cell-surface glycosylation patterns could be determined using various bioanalytic methods. These types of studies have identified a range of transcriptional regulators of enzymes from the MGAT (Zhang et al., 2000; Xu et al., 2011), ST3GAL (Taniguchi et al., 2003), ST6GAL (Taniguchi et al., 2000) and FUCT (Higai et al., 2008) families, among many others. This critical foundational work is too extensive to adequately describe in detail here, but has been well-reviewed elsewhere (Guo and Pierce, 2015; Neelamegham and Mahal, 2016).

In recent years there has been a growing appreciation of the ways that aberrant glycosylation can drive cancer progression (Taylor-Papadimitriou et al., 1999; Saeland et al., 2007; Lin et al., 2014; Beatson et al., 2016; Agrawal et al., 2017). This has led to a specific focus on dissecting how various oncogenes regulate GT expression in cancer cells. One foundational study, for example, demonstrated that overexpression of mutant k-Ras increases 2,6-sialylation of β-integrins in several cancer cell lines. These effects were found to be mediated by downstream transcriptional activation of the ST6GAL1 GT gene (Seales et al., 2003). ST6GAL1 expression is thus likely induced by TFs downstream of k-Ras signaling. More recent studies have since identified such transcriptional regulators, most notably the TF SOX2, which has been found to drive cancer progression through driving expression of both ST6GAL1 (Dorsett et al., 2019) (in ovarian cancer) and ST3GAL1 (Pietrobono et al., 2020) (in metastatic melanoma). These advances in understanding cancer-specific regulation of 2,6-linked sialyloglycan expression were recently well-reviewed (Dorsett et al., 2021). Another key study showed that the TFs c-Myc and CDX2 co-operate to induce expression of selectin ligands in colon cancer cells during epithelial-mesenchymal transition (Sakuma et al., 2012). These changes in glycosylation are mediated by co-ordinated transcriptional regulation of multiple GTs, in particular sialyl and fucosyl-transferases involved in selectin ligand biosynthesis (Sakuma et al., 2012). Separate glycoproteomics-based studies identified another member of this TF family, CDX1, as a key regulator of N-glycan biosynthesis in colorectal carconinoma (CRC) (Holst et al., 2016).

Integration of glycoproteomics and transcriptomic data was also recently used to identify several transcriptional regulators of glycosphingolipid biosynthesis in acute myeloid leukemia (AML) and O-glycan biosynthesis in CRC (Madunić et al., 2021; Wang et al., 2022). Leung et al. also recently used high-resolution mass spectrometry methods to globally map changes in glycosylation induced by overexpression of six common oncogenes (e.g., b-Raf, MEK, AKT) in human cell lines (Leung et al., 2020). These studies revealed significant changes in the biosynthesis of complex N-glycans upon overexpression of several oncogenes. Finally, a study by Möckl and Pedram et al. used super-resolution microscopy to show that k-Ras drives increased “thickness” of the cell-surface glycocalyx, the outer layer of glycosylation that coats all human cells. This effect was found to be mediated by increased expression of the GALNT7 GT enzyme (Möckl et al., 2019). Taken together, these studies illustrate how novel mechanisms of glyco-genomic regulation can be revealed through hypothesis-driven, targeted studies on known oncogenic factors.

**FIGURE 2:**
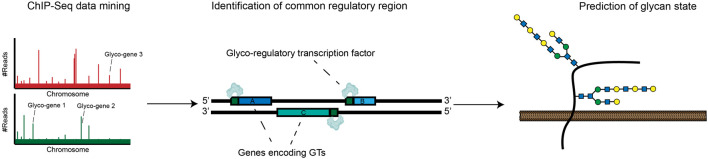
ChIP-Seq approaches can be used to map common promoters bound by specific transcription factors. This data can be incorporated into databases that allow identification of glycan-modifying transcription factors.

A significant complementary trend has been the development of resources that allow for unbiased identification of glyco-regulatory transcription factors. In Chromatin Immunoprecipitation Sequencing (ChIP-seq), TF-DNA complexes are cross-linked and isolated by affinity capture with targeted antibodies. High-throughput sequencing of associated DNA allows for identification of TF-binding sites throughout the genome. Using ChIP-seq, TF-binding motifs can be identified experimentally rather than computationally, reducing the rate of false positive bioinformatic predictions. ChIP-seq is now a mature technology that has been broadly used to produce detailed maps of promoter and enhancer-binding activities for hundreds of cellular transcription factors (Park, 2009; Wu et al., 2015). These results are now readily accessible in online databases, enhancing the throughput with which regulators of glycosylation can be identified. In a recent elegant work, Weiss et al. analyzed ChIP-seq datasets to identify conserved TF-binding sites in the promoter regions of twenty heparan sulfate (HS) biosynthesis genes. This study revealed a specific transcription factor, ZNF263, that binds to ∼50% of gene promoters within this complex biosynthetic pathway. CRISPR/Cas9 knockout of ZNF263 in human cells dramatically increased expression of several enzymes involved in HS biosynthesis and enhanced sulfation of HS chains. This study implies that modulation of ZNF263 expression may be a novel strategy for optimizing cell-based production of heparin, one of the most widely used biopharmaceuticals in the clinic (Weiss et al., 2020). In another recent paper, Groth et al. applied this same type of strategy on a larger scale, mining the Cistrome Cancer Database (which integrates ChIP-Seq data from many cancers) to identify TF-glycogene relationships at a systems level. This novel pipeline identified hundreds of potential glyco-regulatory TFs, creating a rich resource to guide hypothesis generation for the cancer glycoscience community (Groth et al., 2021). These approaches represent generally applicable strategies for discovering regulators of glycan biosynthesis in a range of biological contexts (Figure 2).

**FIGURE 3:**
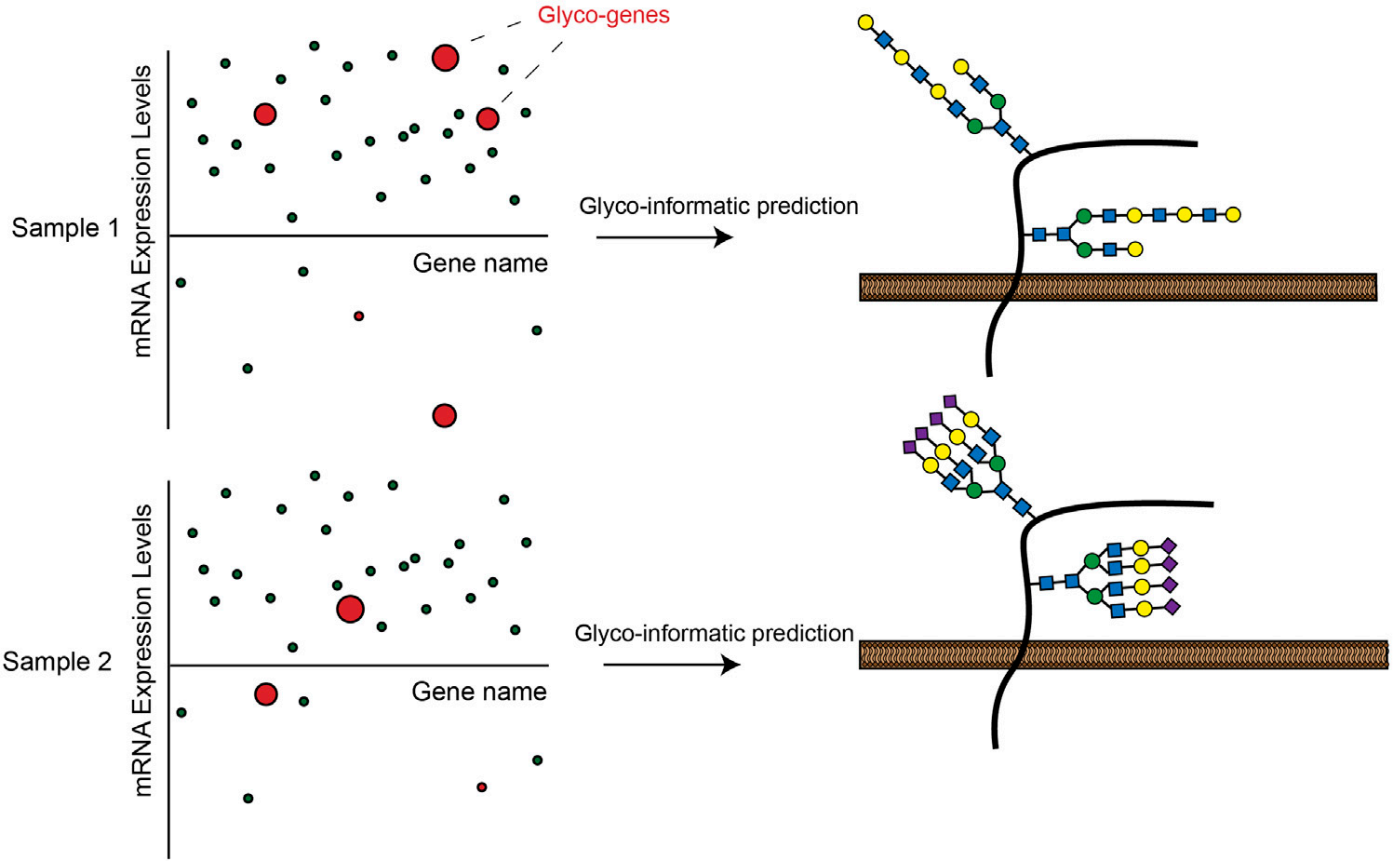
Expression of GT enzymes in RNA-seq datasets can be used to informatically predict a possible cell-surface glycosylation state in a given cell line. These analyses can lead to insights into how glycosylation is differentially regulated across cells and tissues.

Another key development of recent years has been the increasing throughput and declining cost of RNA-seq transcriptomic profiling. This technique allows multiplexed, quantitative measurement of gene expression levels across the protein coding genome. Researchers have used RNA-seq to generate huge quantities of transcriptomic data from a range of cell lines and human tissue samples (Stark et al., 2019). Many of these datasets have since been incorporated into easily searchable online databases such as the Human Protein Atlas (HPA) (Uhlén et al., 2015), The Cancer Genome Atlas Program (TCGA) (Cancer Genome Atlas Research et al., 2013) and the Broad Cancer Cell Line Encyclopedia (Barretina et al., 2012). Several recent studies have used these databases to generate new insights into tissue-specific regulation of cellular glycosylation pathways. In one ground-breaking work, Huang et al. extracted RNA-seq data from the HPA and systematically mapped mRNA expression levels for 950 glycan biosynthesis genes across a selection of human cell lines. The investigators went on to show that integrated transcriptomic analysis of glycogene expression could effectively predict the complement of glycan structures synthesized by a given cell type (Figure 3) (Huang et al. 2021b). In a follow-up study, the investigators applied this tool (GlycoMaple) to study differential expression of glycosaminoglycan (GAG)-related enzymes in various human tissues. This body of work uncovered alterations to GAG biosynthesis in a number of breast cancer subtypes (Huang et al., 2021c). Similar approaches have been used to investigate regulation of glycogene expression in CRC (Wu et al., 2021), cervical cancer (Martinez-Morales et al., 2021), hepatic cancer (Angata et al., 2020) and pluripotent stem cells (Pecori et al., 2021). To facilitate such studies, Thambu et al. also recently developed anexVis, a visualization tool that similarly facilitates transcriptomic analysis of glycan biosynthesis genes (Thambu and Balagurunathan 2022). Systematically dissecting transcriptomic data across a broad range of tissues and disease states creates opportunities for identifying cell and tissue-specific regulators of glycosylation. In one work, for example, Zeng et al. analyzed TCGA data to identify glycosphingolipid biosynthesis genes that are differentially expressed in triple negative breast cancer (TNBC) patients (Zeng et al., 2021). Further dissection of ChIP-seq databases identified the transcription factors AR and GATA3 as putative regulators of glycogene expression in TNBC (Zeng et al., 2021).

Taken together, we expect that these resources will catalyze rapid discovery of new glyco-regulatory mechanisms in the future. In particular, we still see large gains to be made from the integrated study of cancer genomics and glycomics. While the role of oncogenes in driving biosynthesis of specific cancer-promoting glycans is becoming better understood, there has been almost no work studying how loss of tumor suppressor genes affects cell-surface glycosylation. For example, out of the ∼11,000 publications on PubMed that mention the tumor suppressor gene p53, we were not able to identify a single article that examined the role of this protein in regulating glycogene expression or glycosylation. This is a gap in the field we anticipate will be filled in coming years.

### Multi-Layered Regulation: miRNAs and the Cell-Surface Glycome

As discussed above, analyzing the expression of GT-encoding mRNAs can yield important insights into how the glycome is regulated in different cells and tissues. There is some debate, however, as to how consistently mRNA expression data can be used to predict cell-surface glycosylation patterns. Transcriptional activation and repression of glycan biosynthesis genes is clearly one key mechanism that determines which glycan structures will be synthesized by a cell (Neelamegham and Mahal, 2016). However, glycan biosynthesis is also subject to other complex forms of regulation. The expression of an mRNA encoding a specific GT does not guarantee that the mRNA will be translated at high levels or that the corresponding GT will be enzymatically active. Predictions of cell-surface glycosylation states made using transcriptomic data must therefore be carefully calibrated. Hypotheses generated by such approaches should always be validated using state-of-the-art methods for glycan analysis (ideally mass-spectrometry-based glycoproteomics) (Huang et al., 2021b).

The role of micro RNAs (miRNAs) in regulating glycan biosynthesis provides a clear example of this additional complexity. Since the discovery of miRNAs in the early 1990s, evidence has steadily emerged implicating these factors as powerful regulators of gene expression in human cells (Saliminejad et al., 2019). miRNAs are small, non-coding RNAs, typically 22 base pairs in length, that bind to target mRNAs (usually at the 3′-UTR) through complementary base pairing. The typical effect of miRNA binding is to inhibit protein translation or destabilize the target mRNA (Macfarlane and Murphy, 2010). Because miRNA sequences must be complementary to their target mRNA, putative miRNA target sequences can be readily predicted using bioinformatic analysis. In a classic paper, Kurcon et al. informatically identified possible miRNA targets of the miRNA-200 family. The investigators found that miRNAs transcribed from this cluster co-regulate translation of multiple glycogenes, including the GTs B3GLCT, ST3GAL5 and ST6GALNAC5. This “miRNA proxy approach” revealed a linked role for these enzymes in regulating epithelial-mesenchymal transition (EMT) (Kurcon et al., 2015). Multiple other studies have since explored the role of various miRNAs in regulating cancer-associated activity of specific GTs (Gaziel-Sovran et al., 2011; Li et al., 2017; Wu et al., 2017; Ma et al., 2021). This extensive body of work was recently comprehensively reviewed (Thu and Mahal, 2020). In general, the capacity of miRNAs to simultaneously co-regulate translation of many glycan biosynthesis implicates them as “master regulators” of glycan biosynthesis (Figure 1).

It is thus crucial to integrate miRNA expression profiling into systems models that attempt to predict cellular glycosylation states from transcriptomic data. One important aspect of this work is developing better experimental methods for determining exactly which miRNAs regulate a given GT mRNA. Computational methods for miRNA target prediction are known to suffer from high false positive rates (Thu et al., 2021). In one recent paper, Thu et al. developed an elegant high-throughput screening platform (miRFluR) to rapidly identify miRNA clusters that regulate translation of the B3GLCT GT gene (Thu et al., 2021). This study revealed a much narrower set of miRNAs that modulate B3GLCT than would be predicted from computational studies. In the future, we expect that expansion of this approach to a wider set of GT targets will refine our understanding of miRNA-mediated glycome regulation. Even further complexity is also imaginable. Long non-coding RNAs (lncRNAs) for instance, have been shown to play a crucial role in antagonizing miRNA function through competitive inhibition (Xue et al., 2017). One interesting recent study found that a lncRNA with homology to the ST8SIA6 gene (ST8SIA6-AS1) was significantly elevated in breast cancer tissues and promotes invasiveness of breast cancer cell lines (Fang et al., 2020). Findings like this may imply an interesting role for lncRNAs in further tuning glycan biosynthesis, although this hypothesis has yet to be comprehensively explored. These considerations further highlight the need to integrate regulatory RNAs into the next-generation of glyco-informatics models.

### Applications of High-Throughput Genomic Screening in Glycoscience

Functional genetic screening is a powerful strategy for defining genetic regulators of cellular processes and phenotypes. Broadly speaking, functional genetic screening refers to experimental approaches in which the cellular expression of many genes is disrupted in a multiplexed, parallelized manner (Heynen-Genel et al., 2012; Agrotis and Ketteler, 2015). Changes in a given cellular phenotype are then assessed and linked back to specific gene disruption events. Modulation of gene expression can be performed in a variety of different cell models using a diverse array of molecular approaches. Methodologies for assessing and/or selecting for specific phenotypes similarly vary widely. Screens can also be *targeted,* in which a subset of genes of interest are assessed in parallel, or *unbiased*, in which gene function is studied on a genome-wide scale. Until the early-2000s, the model system of choice for large-scale genomic screening studies was budding yeast. Yeast cells are extremely genetically tractable and faithfully recapitulate many evolutionarily conserved aspects of human biology (Enserink, 2012; Adames et al., 2019). However, yeast cells exhibit dramatic differences in glycan structure compared with mammalian cells (Varki A, 2015–2017), which historically limited the applicability of yeast screening techniques to the study of glyco-genomic regulation. Glycoscience researchers have instead often made use of Chinese hamster ovary (CHO) cell collections with defined genetic knockouts in genes encoding GT enzymes. These resources can be effectively used to assess the genetic basis for some glycan-dependent phenotypes, such as cell-surface binding specificities of recombinant lectins (Yang et al., 2015; Nielsen et al., 2018; Nielsen and Wandall, 2022). However, CHO cells are also not a perfect model for human glycosylation, and the number of genes that can be assessed using these methods is relatively small.

The advent and commercialization of RNA interference (RNAi) technology in the early 2000s opened up new possibilities for high-throughput manipulation of gene expression in mammalian cells (Elbashir et al., 2001). These possibilities further expanded with the subsequent development of CRISPR-Cas9 gene editing in the 2010s (Garneau et al., 2010; Jinek et al., 2012). CRISPR-Cas9 allows for genes to be directly disrupted in human cells with unprecedented ease and specificity. Researchers have since adapted CRISPR-Cas9 methods to dissect and engineer cellular glycosylation pathways in living cells. In one foundational work, Stolfa et al. used CRISPR-Cas9 to engineer a small number of cell lines with knockouts in specific glycan biosynthesis pathways (Stolfa et al., 2016). These cell line tools were then used to dissect the contribution of various glycan subtypes to leukocyte adhesion. In a more comprehensive work, CRISPR-Cas9 gene editing was used to generate a panel of isogenic HEK293 cell lines with knockouts in a wide range of key GT enzymes (Narimatsu et al., 2018; Narimatsu et al., 2019). Using this “cell-based glycan array”, recombinant lectins can be rapidly screened to determine specific glycan structures required for binding in living human cells (Büll et al., 2020). One study, for example, applied this approach to identify GTs whose knockout ablates cell-surface binding of Siglecs, immune receptors emerging as targets of interest for cancer immunotherapy (Büll et al., 2021). These methods can also be used in more sophisticated model systems. One recent work used CRISPR-Cas9 to engineer a library of 3D organoid tissues to exhibit various cell-surface glycosylation states (Dabelsteen et al., 2020). This study revealed a key role for specific glycan structures in regulating efficient skin formation (Dabelsteen et al., 2020). Such resources create rich opportunities for array-based functional screening focused on the portion of the genome that encodes glycan biosynthesis genes like GTs.

Soon after its discovery, CRISPR-Cas9 technology was quickly integrated into pooled screening protocols, permitting functional genomics experiments to be conducted on a much larger scale (Shalem et al., 2014; Wang et al., 2014). In CRISPR-based genome-wide screening, a cell line expressing Cas9 is transduced in bulk with a mixed plasmid library containing thousands of sgRNAs. This process creates a pooled, genetically heterogenous cell population in which every cell exhibits a different knockout in a single functional gene. This “cell library” can then be subjected to various types of phenotypic selection, most commonly through exposure to cytotoxic agents or sorting of specific cell populations by fluorescence-activated cell sorting (FACS). NGS is then used to assess how the relative abundance of each sgRNA in the library changes in response to selection. sgRNAs whose abundance shifts significantly in response to selection likely target genes that are functionally linked to the phenotype of interest (Sanjana, 2017; Doench, 2018).

Pooled screening does have some disadvantages relative to arrayed screening approaches. Pooled screens can suffer from high false discovery rates and must be subjected to careful statistical analysis in order to reveal true hits (Sanjana, 2017; Doench, 2018). They also cannot be used to investigate non-cell intrinsic and non-selectable cell phenotypes. However, pooled screening also has some crucial advantages. Most notably, these experiments are: 1) unbiased, in that they assess the function of every gene in the genome, 2) can be completed in a reasonable time frame and 3) use resources commonly found in a typical molecular biology laboratory. Pooled CRISPR screening has thus become an engine of discovery in the life sciences, helping to decode the genetic basis for many cellular phenotypes. Reagents and protocols for genome-wide screening are now broadly available. Recent years have seen the first applications of these powerful methods to the study of glycobiology.

The first of these CRISPR screening studies made use of bacterial toxins (e.g., ricin and Shiga-like toxins) that enter cells through binding to glycan receptors on the cell-surface (Morgens et al., 2017; Tian et al., 2018; Yamaji et al., 2019). Such toxins are highly lethal to human cells, meaning that pooled CRISPR libraries can be easily selected to identify sgRNAs that produce toxin resistance. In several studies, this approach revealed a number of novel genes that are essential for biosynthesis and trafficking of cell-surface glycoconjugates. In addition to recovering known GT enzymes, these screens also implicated some poorly characterized lysosomal (e.g., LAPTM4A) and Golgi (e.g., TMEM165 and TM9SF2) proteins as key regulators of glycan expression (Tian et al., 2018; Yamaji et al., 2019). These screens thus suggest novel mechanisms of glycan synthesis regulation within the secretory pathway, providing interesting directions for future research.

Subsequent work has used CRISPR-Cas9 screening to probe more specific glycan biosynthesis pathways. In one study, cells were infected with a genome-wide library of sgRNAs and incubated with a fluorescently-labelled ligand that binds selectively to HS. Cell sorting techniques (FACS) were then used to isolate cells exhibiting reduced binding. Subsequent sgRNA sequencing revealed the KDM2B gene as a top hit. In follow-up work, KDM2B inactivation was shown to inhibit expression of multiple sulfotransferase enzymes and upregulate expression of sulfatases, implicating this TF as a novel “master regulator” of HS biosynthesis (Weiss et al., 2021). Other studies have applied a similar FACS-based screening approach to identify regulators of glycosylation in cancer. In one recent work, the investigators used a genome-wide CRISPR interference (CRISPRi) screening strategy to identify genes that reduce binding of recombinant Siglec receptors to the surface of leukemia cells (Wisnovsky et al., 2021). This approach revealed a cluster of genes including 1) the GTs C1GALT1 and ST6GALNAC1 and 2) the cell-surface protein CD43 to be drivers of Siglec-7 ligand expression. Further investigation revealed that Siglec-7 binds to a distinct glycopeptide motif containing clusters of disialylated O-linked glycans (Wisnovsky et al., 2021). The GTs identified in the screen biosynthesize these glycans, while the CD43 protein acts as a scaffold that presents these carbohydrates at an unusually high spatial density. The combination of these two factors mediates selective Siglec-7 binding. These types of studies exemplify how genomic screening can be used to dissect the complex polygenetic circuits that regulate glycan ligand expression.

**FIGURE 4:**
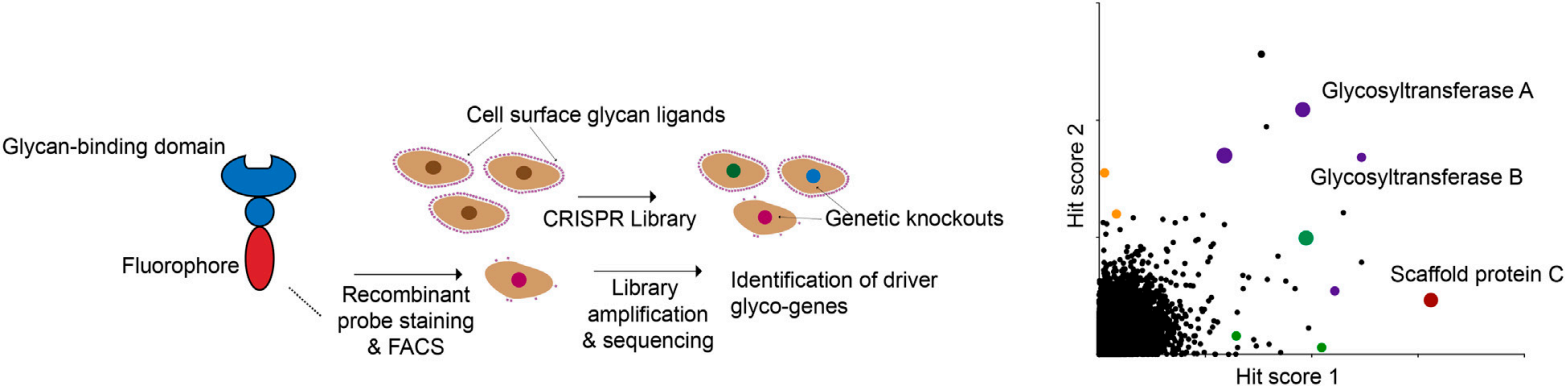
A modular CRISPR screening strategy for identifying genes that regulate binding of a lectin to the surface of living cells. The abundance and ready availability of different glycan-binding proteins that can be adapted for this assay make this a general approach to glyco-genomics research.

These proof-of-principle studies open up numerous possibilities for the future. Decades of glycobiology research have uncovered dozens of lectins with various specificities for different classes of glycans (Bojar et al., 2022). In principle, any of these lectins (many of which are commercially available in large quantities) can be easily used to identify genetic regulators of specific glycans using FACS-based screening (Figure 4). In anticipation of these possibilities, CRISPR screening resources targeted specifically at glycobiology users are now being developed. A recent study, for example, described the generation of a small, pooled CRISPR library targeting 347 known glycan biosynthesis genes (Zhu et al., 2021). The investigators used this library to identify genes essential for the cell-surface binding of several known lectins. Using a compact sgRNA library allows for screens to be performed quickly and at low cost, while still permitting multiplexed functional annotation of glycosylation-related genes. While genome-wide libraries will still be the tool of choice when an unbiased approach is needed, these types of targeted resources will also be of great use to the glycobiology community.

### Dissecting Glycomic Heterogeneity: Glycan Analysis at Single-Cell Resolution

Finally, the most recent interesting trend to emerge in glyco-genomics has been the application of single cell profiling methods to the study of glycobiology. Cell-surface glycosylation notoriously displays significant biochemical heterogeneity. For example, glycoproteomics studies performed on bulk cell mixtures frequently discover that many different glycan structures can be appended to a single glycosylation site (Oliveira et al., 2021). In part, these findings likely reflect differential regulation of glycan biosynthesis by different cells within complex populations. The recent development of techniques like scRNA-seq now offer new opportunities for determining the genetic basis for these distinct cellular “glyco-states”. In scRNA-seq, single cell suspensions are disassociated from cultured cells or tissue and physically separated using a variety of methods (Nayak and Hasija, 2021). Microfluidics-based systems, in which separate cells are captured within individual microfluidic droplets, have gained particular popularity in recent years (Nayak and Hasija, 2021). Individual cells are frequently sorted *via* FACS to further isolate cells exhibiting expression of specific cell-surface markers. mRNA from individual cells is then amplified by RT-PCR and sequenced using NGS. These techniques can be used to build detailed gene expression maps for thousands of individual cells, providing a high-resolution look into the heterogeneity of gene expression within a cell population.

One important recent study pioneered a new technique (SUGAR-Seq) that integrates scRNA-seq and glycomics analysis. In this approach, tumor-infiltrating T-cells (TILs) were isolated from primary tumors and labelled with a lectin (L-Pha) that binds to complex branched N-glycans (Kearney et al., 2021). Single TILs were then sorted based on L-Pha staining and analyzed by scRNA-seq to reveal differences in glycosylation amongst different TIL subsets. This study revealed several insights. Most notably, it was found that cells exhibiting regulatory and exhausted gene expression signatures were more densely N-glycosylated than active memory or effector T-cells. Follow-up glycoproteomic and lectin proximity labelling studies also indicated changes in glycosylation of immune checkpoint receptors and ligands commonly expressed on T-cells. These findings may thus have important implications for understanding T-cell regulation in cancer and for the design of new immune checkpoint inhibitors.

Another set of recently-published protocols also applied single cell profiling to study glycosylation in human induced pluripotent stem cells (Minoshima et al., 2021; Odaka et al., 2022). Single-cell suspensions were stained with several lectins, sorted by FACS and analyzed by scRNA-seq. These studies revealed that differences in glycosylation were associated with differential expression of genes associated with neural differentiation (Minoshima et al., 2021). The protocol used for this work was also separately described in a detailed methods article (Odaka et al., 2022). As with CRISPR screening, the availability of a wide range of lectins for conducting these types of studies creates a range of exciting possibilities. We thus anticipate that “single cell glycomics” will continue to develop in the future.

### Future Perspectives: What’s Next for Glyco-Genomics?

In this review, we hope we have adequately summarized some of the most significant recent advances at the interface of genomics and glycomics. In the future, we believe continued progress in this area will allow researchers to build sophisticated, systems-oriented models of how glycosylation is regulated in living cells. In addition to the perspectives we have already offered, we see several aspects of this research that have significant potential for growth.

As has been discussed, transcriptomics datasets can now be harnessed to predict cellular glycosylation patterns with amazing speed and considerable accuracy. However, it is clear that quantitating GT expression levels is not sufficient to analyze the glycome in its full complexity. Systems analyses of cellular glycosylation must account for other mechanisms by which glycosylation may be regulated. miRNA-mediated regulation is clearly relevant, but other areas remain chronically underexplored. Several CRISPR screening studies, for example, have identified specific ER and Golgi-relevant chaperones as key players in glycan biosynthesis (Tian et al., 2018; Yamaji et al., 2019; Wisnovsky et al., 2021). The tissue-specific expression and/or function of these types of molecules has yet to be analyzed in a systematic way. Another gap in the field is understanding the post-translational regulation of glycan biosynthesis. To take one example, many GTs and other Golgi-localized proteins have been found to be phosphorylated in living cells (Yu and Bieberich, 2001; Bergeron et al., 2017). However, there has not been significant recent exploration of how these phospho-sites are dynamically altered (e.g., by cancer-associated kinases) or how they functionally modulate GT activity. To develop truly comprehensive models of how glycosylation is regulated, we will also need to study these types of mechanisms and closely integrate them with insights from genomics studies.

As discussed above, we view the application of single-cell profiling techniques as a leading edge of this research area. In addition to scRNA-seq, other optimized technologies like single cell ATAC-seq (for analyzing epigenomic states) and Perturb-seq (integrating functional genetic screening with scRNA-seq) are ready to be applied to questions in glycobiology (Dixit et al., 2016; Fang et al., 2021). Current single cell profiling methods do face some technical and financial limits on sequencing depth (Haque et al., 2017; Nayak and Hasija, 2021). These factors can make it difficult to accurately profile expression of transcripts with low copy numbers. In the context of glyco-genomics, this means that integrated expression analysis of glycan biosynthesis genes (which is now routine for bulk RNA-seq) is likely not yet feasible at single cell resolution. However, single cell sequencing technologies are advancing at a rapid clip, and we may well see new technical breakthroughs that solve some of these challenges.

Finally, the incredible proliferation of different CRISPR-Cas9-based gene manipulation technologies is poised to create new breakthroughs in glycoscience. We conclude by suggesting several novel applications. Firstly, in CRISPR activation (CRISPRa) screening, sgRNA-mediated overexpression of target genes is induced by recruitment of a dCas9-VPR fusion protein (VPR is a transcriptional activator). This technique allows for gain-of-function gene overexpression screens to be easily performed in human cell lines. CRISPRa can induce expression of genes that are not normally active in most model cell lines, allowing for broad functional annotation of tissue and context-specific genetic factors to be performed in a single cell type (Maeder et al., 2013; Gilbert et al., 2014). In future, we envision that CRISPRa screening could be usefully applied to dissect functions of genetically redundant, complementary glycogenes that would be missed by traditional loss-of-function approaches. Secondly, in multiplexed CRISPR screening, multiple sgRNAs are co-expressed from the same promoter, permitting simultaneous knockout of multiple genes in the same cell. Construction of unique barcoded combinatorial libraries allows for rapid screening of pairwise genetic interactions (Wong et al., 2016; Han et al., 2017). These types of techniques are ideal for studying the complex, polygenetic networks that regulate glycosylation. Finally, CRISPR screening in organoid cultures (Han et al., 2020), primary cells (Shifrut et al., 2018) and *in vivo* (Huang H. et al., 2021) also offer new options for dissecting glycan regulation in sophisticated model systems. The possibilities here are near limitless, and we are excited to see what new frontiers in glyco-genomics emerge from this type of research.
